# Enhanced real-time global ionospheric maps using machine learning

**DOI:** 10.1007/s10291-025-01858-0

**Published:** 2025-05-12

**Authors:** Marcel Iten, Shuyin Mao, Yuanxin Pan, Benedikt Soja

**Affiliations:** ETH Zurich, Zurich, Switzerland

## Abstract

Global ionospheric maps (GIM) are commonly used ionospheric products in high-precision Global Navigation Satellite System (GNSS) applications. To meet the increasing demand for real-time (RT) applications, the International GNSS Service (IGS) officially started a real-time service in 2013. One of the tasks of the real-time service is the calculation of real-time GIMs. However, the accuracy of current real-time GIMs is still significantly worse than that of the final GIMs, which are the most accurate ionospheric products but have a latency of several days. The IGS RT GIMs exhibit an RMSE of around 3.5–5.5 total electron content units (TECU) compared to the final GIMs. This study focuses on improving the accuracy of existing real-time GIMs through machine learning (ML) approaches, specifically convolutional neural networks (CNN) and conditional generative adversarial networks (cGAN). We apply our method to the IGS combined real-time GIMs and to Universitat Politècnica de Catalunya (UPC) GIMs. We consider over 130’000 pairs of real-time and final GIMs. Over a 3.5-month test period, the proposed approach shows promising results with a reduction of more than 30% in mean absolute error for the real-time GIMs. Especially for regions with high VTEC values, we find a significant improvement of nearly 50%. The ML-enhanced real-time GIMs also exhibit improved positioning performance for single-frequency GNSS positioning with reductions in the 3D error up to 21 cm. Overall, our proposed method demonstrates great potential in generating more accurate and refined real-time GIMs.

## Introduction

The ionosphere is an atmospheric layer ranging from a height of about 60 km to 2000 km above the Earth’s surface (Bilitza et al. 2022). It leads to a refraction effect that changes the travel speed of Global Navigation Satellite System (GNSS) signals, which can lead to significant positioning errors if not considered properly. The local state of the ionosphere is usually expressed in total electron content (TEC), which is the number of electrons in a volume with a 1 m$^2$ cross-section along the signal path. TEC is expressed in TEC units (TECU) where 1 TECU = 10$^{16}$ electrons/m$^2$, which is equivalent to approximately 16 cm of range error at the L1 frequency of Global Positioning System (GPS) (Dyrud et al. 2008). The GNSS satellites, combined with globally distributed ground receivers, have facilitated continuous monitoring of the ionospheric conditions. In 1998, the International GNSS Service (IGS) Ionosphere Working Group was established and started with the routine generation of the combined Vertical Total Electron Content (VTEC) models, so-called Global Ionospheric Maps (GIMs), which describe the state of the ionosphere (Hernández-Pajares et al. 2009). The VTEC maps are distributed in the unified IONosphere Map EXchange (IONEX) format (Schaer et al. 1998). Different analysis centers contribute to the generation of GIMs, which typically exhibit a spatial resolution of $2.5^\circ$ in latitude and $5^\circ$ in longitude (Liu et al. 2021b). GIMs are currently available from the IGS as products of three different latencies, namely the predicted, the rapid and the final GIMs (Liu et al. 2021b). The predicted solutions are available one or two days prior, rapid solutions are available with a latency of 24 h and the final solutions with a latency of approximately 11 days (Noll 2010). Currently, eight IGS Ionosphere Associate Analysis Centers (IAACs) produce the mentioned GIMs on a daily basis. From the products of these contributing IAACs, the Ionosphere Product Coordinator computes the official IGS combined products (Noll 2010). While the predicted GIMs can be used to extract ionospheric VTEC information in advance, they reveal a limited accuracy (Liu et al. 2021b). Final GIMs, featuring the highest accuracy, are not available for real-time applications due to their latency (Ren et al. 2019). To meet the increasing need for real-time positioning and its applications, the IGS formed the Real-Time Working Group (RTWG) in 2001 with an official start of the real-time service (RTS) in 2013. Among other real-time products like satellite orbits and clocks, also GIMs are published through this service. Several analysis centers contribute with a continuous generation of their individual real-time GIMs. The IGS further provides the IGS combined real-time GIM, which is a weighted combination of the GIMs from individual analysis centers and has a latency of a few minutes (Liu et al. 2021b). Strictly speaking, real-time GIMs are a near real-time product but are commonly referred to as real-time. Real-time GIMs enhance the accuracy of single-frequency positioning by delivering real-time ionospheric corrections. Additionally, they play a crucial role in monitoring space weather and providing early warnings for potential natural hazards (Zhang et al. 2019; Tariq et al. 2019; Jin and Song 2023).

The comparison of different real-time GIM products with the IGS final GIM shows that the real-time products have significantly lower accuracy. The agreement also depends on the solar activity, with the consistency of real-time product degrading with increasing solar activity. (Liu et al. 2023) found that in 2022, a year with increasing solar activity, the IGS real-time GIMs had a root mean square (RMS) of 3.33 TECU compared to the IGS final GIMs. Daily biases of different real-time products compared to the IGS final GIM were within 4 TECU and largest at low-latitude regions with higher VTEC values. The limited number of GNSS stations in the southern hemisphere contributing to the generation of the VTEC maps is also likely a reason for a hemisphere asymmetry leading to a worse accuracy of GIMs in the southern hemisphere (Li et al. 2020). For real-time applications, it is desirable to further improve the accuracy of the real-time GIMs and narrow the gap to the final GIMs.

In recent years, machine learning (ML) has evolved as a powerful tool for various scientific applications. It also found its way to the field of GNSS and space geodesy. (Ji et al. 2020) applied deep learning in the form of conditional Generative Adversarial Networks (cGANs) to improve the quality of International Reference Ionosphere (IRI) TEC maps. They were able to reduce the RMS from 18.7 TECU to 13.3 TECU during high solar activity periods, and from 9.7 TECU to 7.3 TECU during low solar activity periods. They found that the ionospheric peak structures were especially improved compared to the IRI TEC maps. (Liu et al. 2022) used ML in the form of a convolutional long short-term memory (convLSTM) neural network to forecast GIMs up to 24 h. They investigated a residual prediction where the model learns to predict the residuals between the subsequent TEC maps and a direct prediction model which predicts the next TEC map from the previous one. For their work, they find the best performance with a residual prediction strategy and an *L*1 loss function. Generative Adversarial Networks have also been used to complete missing data gaps in GIMs in (Yang et al. 2022). To account for potentially missing TEC data, they removed certain patches from the GIMs and trained the model to predict the missing patches. The GAN exhibited its generative capabilities by successfully predicting the data gaps, reaching differences of around 5 TECU around the ionospheric peak structure. cGANs have also been used in (Lee et al. 2021) for one-day forecasting of global TEC maps. Their approach works by only using daily TEC maps and one-day difference maps as input for the model. With this forecast model, they achieved an improved performance compared to the one-day CODE prediction model (Schaer 1999). A feed-forward neural network (NN) was used in (Mao et al. 2024) to perform spatial interpolation of VTEC values. The created NN GIM resulted in improved positioning accuracy compared to the Chinese Academy of Sciences (CAS) GIM.

Our study explores the potential of ML in enhancing the accuracy of real-time GIMs. Since the GIMs we use are two-dimensional data, they can be treated like images. Our problem formulation is similar to that of (Ji et al. 2020) where cGANs exhibited great performance. A one-to-one mapping has the advantage of not being affected by data gaps, unlike approaches that rely on sequential inputs. Transformers, which are powerful tools for processing sequential data typically require large datasets. However, this is contradictory in our case, as real-time GIMs have only been available since 2019. Therefore, we investigate a U-Net (Ronneberger et al. 2015) in both a traditional Convolutional Neural Network (CNN) approach and in a cGAN (Mirza and Osindero 2014) approach to transform the classical real-time GIM to an ML real-time GIM with improved accuracy. To learn this mapping, we train the model with real-time GIMs as input and final GIMs as target.

This paper is organized as follows: The ionospheric data used in this study is described in Sect. 2. The CNN and cGAN methods are explained in Sect. 3. Results, including comparisons with final GIMs and satellite altimetry, as well as an application in single-point positioning, are presented in Sect. 4. Finally, the conclusion and outlook are provided in Sect. 5.

## Data

### GIMs

In this study, we use real-time GIMs and final GIMs from two different providers, namely IGS and UPC (UADG products) due to their high quality (Liu et al. 2021b). This choice makes it possible to investigate whether the proposed method can be applied to different datasets.

**Fig. 1:**
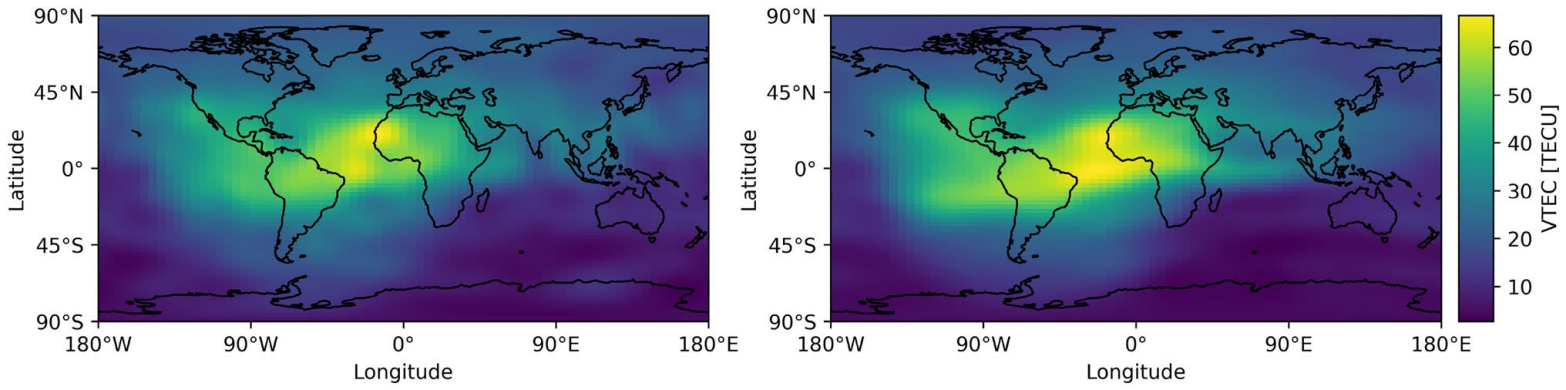
Example of IGS real-time (left) and final (right) global ionospheric map from 2023-07-14 16:40:00 UT

Our datasets consist of real-time and final GIM pairs. Figure 1 shows a visualization of a real-time and final GIM pair from IGS. The number of such pairs is limited by the availability of the real-time products where the UPC archive reaches back to late 2019 and the IGS archive to the beginning of 2021. IGS real-time GIMs are available with a temporal resolution of 20 min whereas UPC real-time GIMs have a temporal resolution of 15 min. To create our dataset, we need to pair real-time GIMs with their corresponding final GIMs. Due to the lower temporal resolution of the final GIMs (sampled by 2 h), we interpolate them to match the real-time epochs. We follow the approach of (Schaer et al. 1998) to interpolate between consecutive rotated TEC maps. This interpolation of a TEC value *E* at a geocentric latitude $\beta$, longitude $\lambda$ and universal time *t*, given TEC maps $E_i=E(T_i), i = 1,2,...,n$ can be formulated as follows:

1 $$\begin{aligned} E(\beta ,\lambda ,t) = \frac{T_{i+1}-t}{T_{i+1}-T_i}E_i(\beta ,\lambda '_i) + \frac{t-T_i}{T_{i+1}-T_i}E_{i+1}(\beta ,\lambda '_{i+1}), \end{aligned}$$

with $T_i \le t < T_{i+1}$ and $\lambda '_i = \lambda +(t-T_i)$ where units need to be consistent between angles and times. This interpolation method is very common; however, it should be noted that a temporal resolution of two hours for a GIM product might be less accurate in regions with high temporal ionospheric variation (Liu et al. 2021a).

We constructed three datasets (see Table 1) based on different real-time and final GIM combinations. This is to demonstrate that the proposed method can generally be applied to specific selections of real-time and final products, even from different providers.

**Table 1:** Description of datasets constructed for evaluation, showing combinations of real-time and final GIMs, along with corresponding number of data pairs and temporal coverage

Dataset name	Real-time GIM (Input)	Final GIM (Target)	# Data pairs	First epoch	Last epoch
IGS-IGS	IGS	IGS	70054	2021-01-24	2023-10-14
UPC-IGS	UPC	IGS	135965	2019-10-09	2023-10-14
UPC-UPC	UPC	UPC	137671	2019-10-09	2023-10-14

**Fig. 2:**
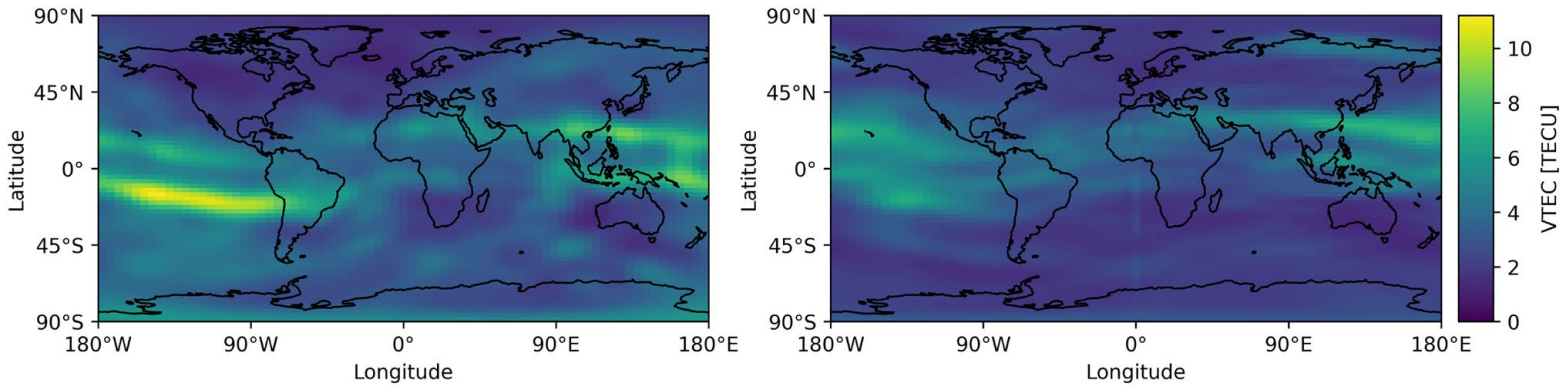
Mean absolute differences of IGS (left) and UPC real-time GIMs (right) with respect to the IGS final GIMs for the test period 2023-07-01 to 2023-10-14

Specifically, we build two datasets based on the IGS final GIMs, once using IGS real-time GIMs and once UPC real-time GIMs as input. A third dataset is constructed using UPC GIMs for the real-time and final part. The IGS-IGS (i.e., IGS real-time $\rightarrow$ IGS final) dataset comprises approximately 70’000 data pairs. The UPC-IGS and UPC-UPC datasets are larger with approximately 136’000 data samples each. The difference in the number of samples is due to the different availability and temporal resolution of real-time GIMs. Figure 2 shows the averaged absolute differences of the IGS and UPC real-time GIMs compared to the IGS final GIM. It highlights that, as mentioned before, the real-time products exhibit significant differences compared to the final products. This is particularly evident in the IGS real-time product, where pronounced differences can be seen in the low latitudes.

**Fig. 3:**
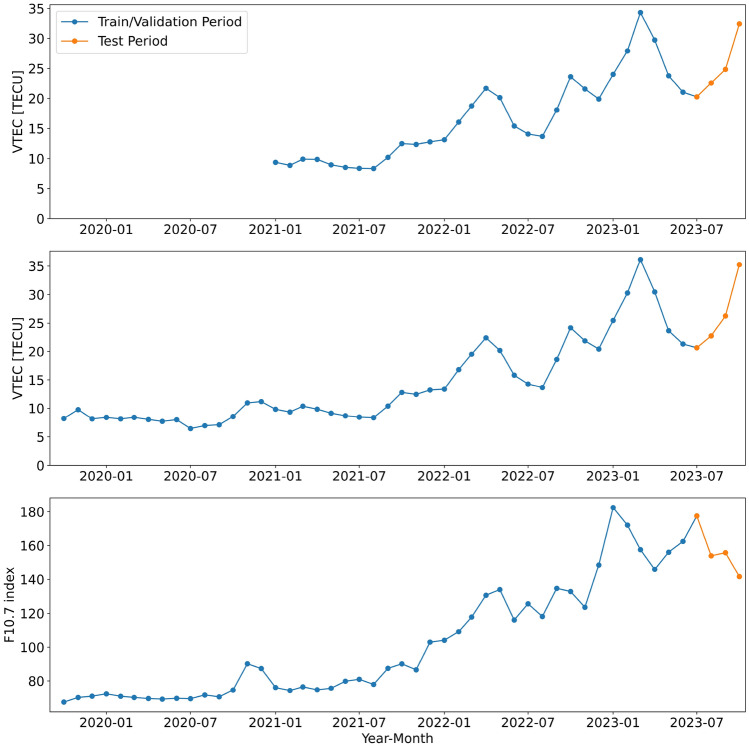
Monthly global mean VTEC values of the IGS real-time GIMs (top) and the UPC real-time GIMs (middle). The mean VTEC values exhibit a correlation with the monthly mean F10.7 solar flux index (bottom). The data represents the values for the train/validation (blue) and test (orange) period

**Fig. 4:**
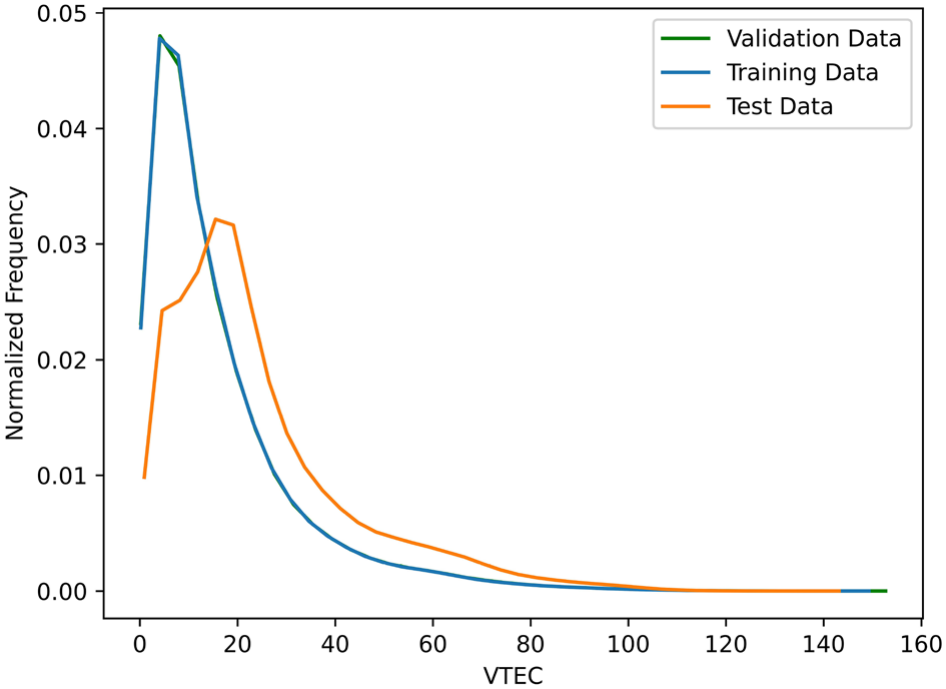
Distribution of the VTEC values in the IGS-IGS dataset. Training and validation data are overlapping. The test dataset is biased towards higher VTEC values

We divide our datasets into training, validation, and test sets. To account for a later application in an operational setting, we choose the test data to cover the last period of the whole dataset. This approach ensures that the model is trained completely on prior data and does not get any information from later epochs, which is typically the case in an operational real-time setup. The test data covers approximately 3.5 months ranging from 2023-07-01 to 2023-10-14. The rest of the dataset is randomly split into 90% training and 10% validation data based on the epochs. It is important to note that the training and validation sets cover different solar activities than the test set (see Fig. 3). The solar activity described by the F10.7 index, representing radio emissions at 10.7 cm wavelength, ranges from low values for October 2019 to high values by the end of 2023. Due to the limited availability of the real-time GIMs, not covering a complete solar cycle, the test set falls into a period of high solar activity while the training and validation set cover periods of both low and high solar activity. The data distribution of the test set thus differs from that of the training and validation sets (see Fig. 4), posing a challenge for machine learning models.

### Altimetry and GNSS data

**Fig. 5:**
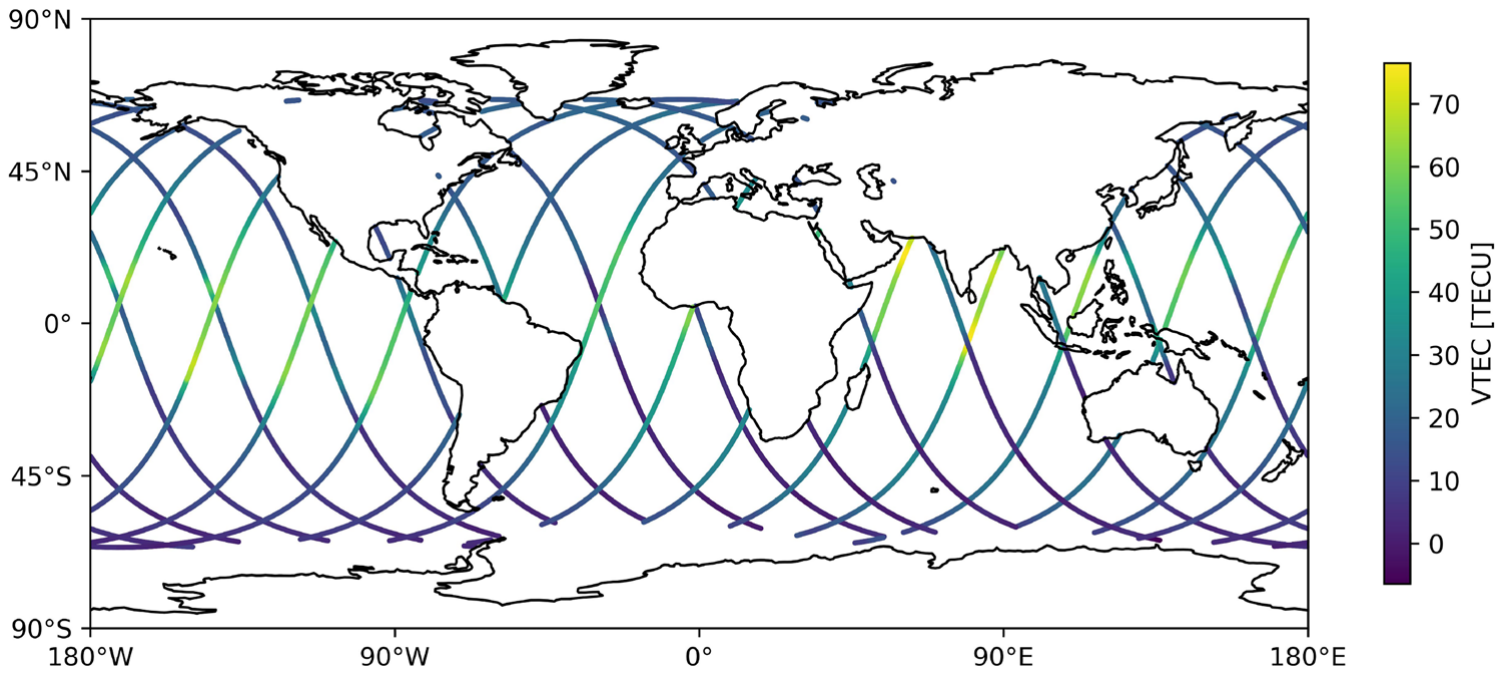
Ground track of Jason-3 and observed VTEC for 2023-08-10

It is common to validate GIM products using independent external satellite altimetry data, which provides VTEC estimations over the oceans (Wielgosz et al. 2021; Ren et al. 2019; Li et al. 2020, 2019). In this study, we use VTEC values from the Jason-3 satellite altimetry mission. The Jason-3 satellite was launched in 2016 and is still operational today. It is designed as follow-on mission to Jason-2 to continue the core satellite altimetry measurements for physical oceanography. It has a repeat time of 9.9 days to ensure the continuity of the global sea level record and flies at an altitude of approximately 1360 km (Vaze et al. 2010). Due to Jason-3’s lower orbit height compared to GNSS satellites, it cannot sound the same part of the plasmasphere and is expected to obtain systematically lower VTEC values, considering the contribution of plasmaspheric electron content (Lee et al. 2013). Still, it can be used for evaluation by looking at the bias between Jason-3 and different GIM products, as well as the standard deviation of the residuals after correcting for such a bias (Liu et al. 2021b). Figure 5 shows an example of the daily trajectories of the Jason-3 satellite along with the corresponding VTEC observations. These VTEC values can then be compared with those from GIM products as described in section 3.5.2.

**Fig. 6:**
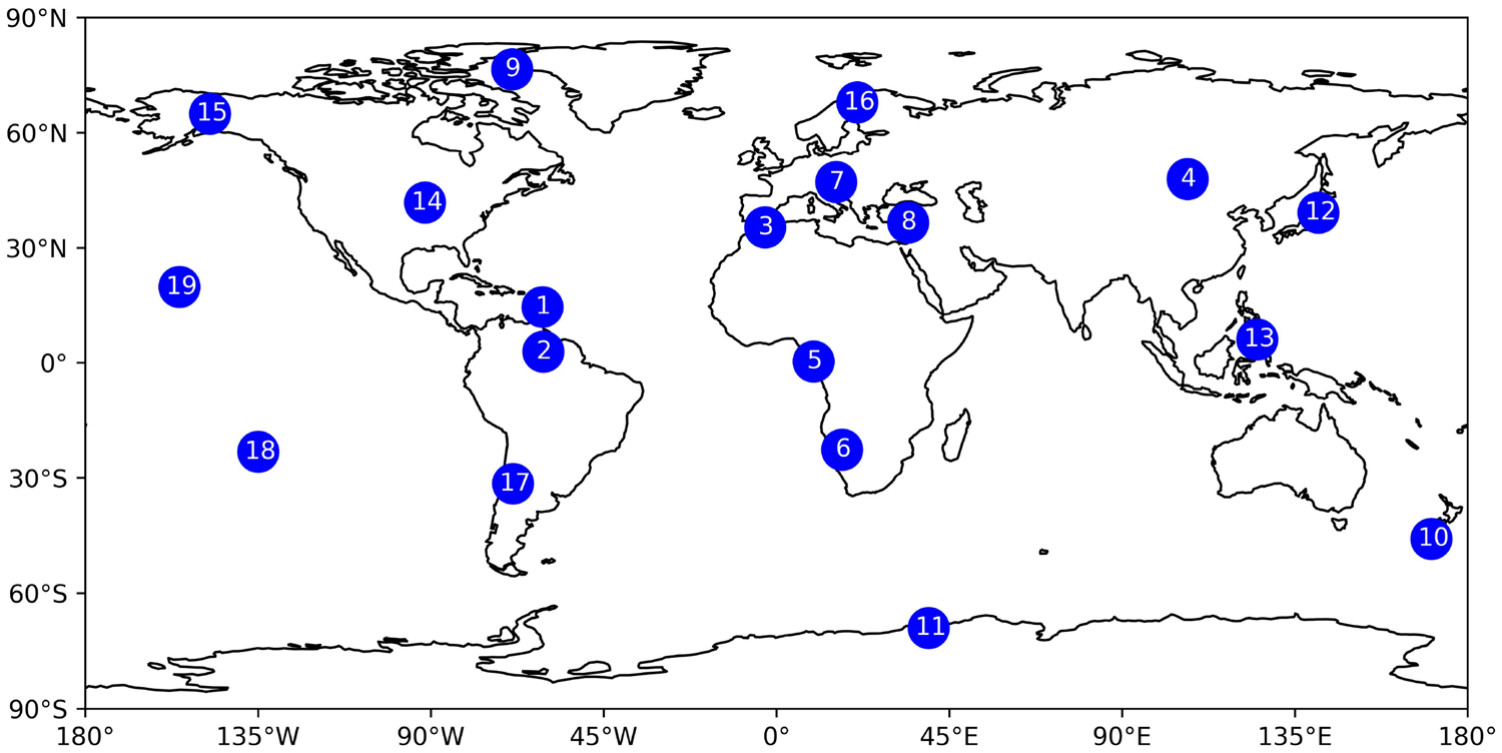
Locations of IGS stations used for SPP evaluation

Further we perform single point positioning (SPP, see Sect. 3.5.3) on 19 globally distributed IGS GNSS stations shown in Fig. 6. Since the GIMs represent global VTEC, evaluating them at stations of different latitudes and longitudes provides a more comprehensive assessment of the overall performance. The names and exact locations of the stations used are listed in Table 14 of the appendix A.

## Methods

ML demonstrates remarkable capabilities in learning from extensive datasets, capturing complex patterns within the data. This makes it especially useful for our task, which involves over 130,000 samples with both spatial and temporal information. Since our input and target data are two-dimensional objects, we investigate two different ML methods capable of handling image-like data, namely cGAN and CNN. As mentioned in Sect. 1, GANs have been successfully used in similar tasks related to the ionosphere and have shown promising performance. However, they can be challenging to train (Sinha et al. 2020). The classical CNN has also demonstrated remarkable capabilities in tasks related to image processing. The detailed usage of both methods will be explained in the following two sections.

### CNN method

The CNN method consists of one model that we call generator to highlight the parallels to the cGAN. The generator learns the mapping from some input data *x* to some target data *y*. Additionally, noise *z* can be added to the input. This mapping can then be described as $G: \{x,z\} \rightarrow y$. In our case, *x* are the real-time GIMs plus additional features and *y* are the final GIMs. We use a U-Net (Ronneberger et al. 2015) architecture implementation from Segmentation Model Pytorch (SMP) (Iakubovskii 2019) as generator *G*, which is capable of taking an image of a certain size as input and outputting an image of the same size. We resize our input data to a 96x96 shape by reflection padding. A ResNet18 (He et al. 2015) is selected as the encoder for the U-Net. Additionally, we modify the last layer of the U-Net and apply a Rectified Linear Unit (ReLU) (Agarap 2019) activation function to ensure that the model does not output any negative VTEC values. The objective is to minimize the differences between the final GIM and the created ML GIMs by the generator. We use *L*1 loss and define the objective functions as

2 $$\begin{aligned} {\mathcal {L}}_{CNN} = {\mathcal {L}}_{L1}(G) = \Vert y-G(x,z)\Vert _1 \end{aligned}$$

**Fig. 7:**
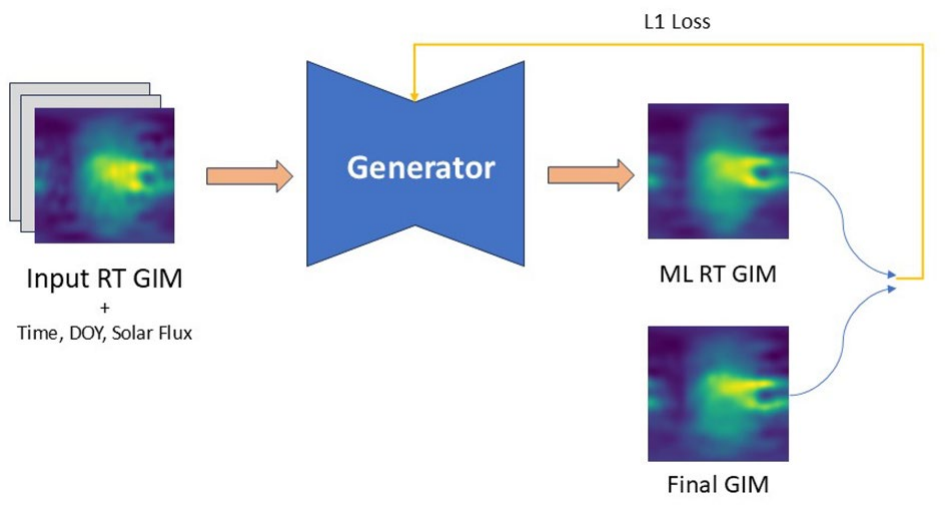
Visualization of the CNN method. The generator tries to generate output with minimal *L*1 loss compared to final GIM map

We also tested *L*2 loss but did not observe any significant changes. Therefore, selected *L*1 loss since it encourages less blurring (Isola et al. 2018). A visualization of the CNN method is shown in Fig. 7.

### cGAN method

Classical GANs learn the mapping from a random noise vector *z* to an output image *y*, which can be described as $G: z \rightarrow y$ (Goodfellow et al. 2014). For our purpose, we condition the generator and discriminator on additional data. For this conditional GAN, the mapping is different in a way that both observed data *x* and noise *z* are mapped to *y*, $G: \{x,z\} \rightarrow y$ (Isola et al. 2018).

We create and implement an ML setup in pytorch (Paszke et al. 2019) based on the cGAN architecture. The cGAN consists of two models: a generative model called generator, and a discriminative model called discriminator. Given training data and noise as input, the generator is trained to generate ’fake outputs’ with the intention of deceiving the discriminator. The discriminator tries to determine whether the given sample is from the training data or is generated by the generator (Goodfellow et al. 2014; Ji et al. 2020).

We use the U-Net introduced in 3.1 as generator *G*. The discriminator *D* is implemented as a sequence of convolution layers with a scalar value output representing the probability of the sample belonging to the training data.

**Fig. 8:**
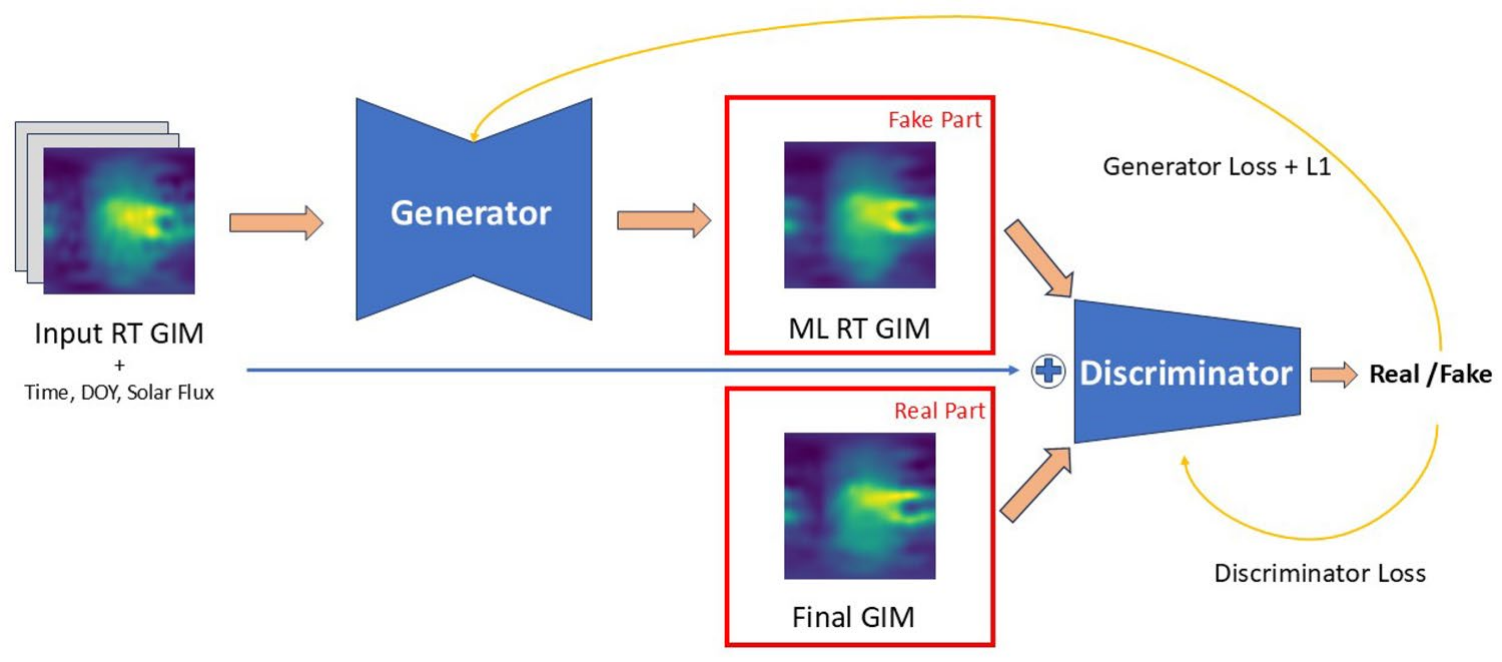
Visualization of the cGAN architecture. The generator learns to deceive the discriminator. The discriminator learns to distinguish real and fake samples

The generator *G*(*x*, *z*) learns the mapping from the real-time GIM *x* with additional features (see Sect. 3.3 for more information) and noise *z* to the ground truth *y*. We define the objective function of the cGAN as follows:

3 $$\begin{aligned} {\mathcal {L}}_{cGAN}(G,D) = \log (D(x,y)) + \log (1-D(x,G(x,z)), \end{aligned}$$

where G tries to minimize this objective and D tries to maximize it (see eq.(4)). Since the overall goal is to minimize the differences between the final GIM and the created GIM by ML, we also add an *L*1 loss to the objective, as well as an additional parameter $\lambda$ to control the relative weight between the cGAN and L1 loss. This results in the final loss function

4 $$\begin{aligned} G^* = \text {arg} \, \underset{G}{\text {min}} \, \underset{D}{\text {max}} \, {\mathcal {L}}_{cGAN}(G,D)\cdot \frac{1}{\lambda } + {\mathcal {L}}_{L1}(G) \end{aligned}$$

where we experimentally test a various values for $\lambda$ and observe only minor changes in performance. For the final results, we select $\lambda = 2500$ as a suitable choice. Our generator consists of 11.3 and the discriminator of 2.8 million parameters. The entire architecture of the cGAN applied in this work is depicted in Fig. 8.

### Additional features and noise

We utilize not only the real-time GIM as input for the ML model but also additional features. We include monthly solar radio flux index (F10.7) from National Oceanic and Atmospheric Administration (NOAA) Space Weather Prediction Center, providing information about current solar activity levels. Further more, we incorporate seconds of the day and day of the year as input features to capture both diurnal and seasonal variations. For this purpose, they are simply scaled into a range from 0 to 1. We experimented with introducing different levels of white noise to the input real-time GIM but observed no significant performance changes compared to the scenario without noise. For the final models, no noise is used leading to $z=0$.

### Training and model selection

To train both CNN and cGAN models, we use a batch size of 64 and a learning rate of 0.0005 together with the ADAM optimizer, and neither dropout nor weight decay is applied. For the cGAN training, this learning rate in combination with the chosen $\lambda$ leads to stable adversarial training. In addition, the added L1 loss to the cGAN objective eq.(3) helps the model to not only produce a single output since this would be penalized (Isola et al. 2018). For each of the three created datasets, we train five models and aggregate them into an ensemble by averaging their output. During the training process, the five models are initialized with different seeds. For each of the five models, the training data is randomly split into training and validation data. We evaluate the *L*1 loss on the validation set for each training epoch and select the model state from the epoch with the lowest loss.

### Evaluation methods

To quantify the impact of machine learning on the accuracy of the real-time GIMs, we perform three evaluations with the data from the test period. First, an internal evaluation is done by directly comparing the agreement of the real-time GIMs and the machine learning output with the final GIM product on the test set. Then, we conduct an external evaluation using VTEC observations from the Jason-3 satellite altimetry mission as reference, assessing the GIM performance over ocean regions. To compare the different GIMs, we calculate the daily standard deviation (STD) between the VTEC values from altimetry and the GIMs. Finally, we perform a single point positioning (SPP) test to estimate the impact of the different GIMs on positioning applications.

#### Internal GIM evaluation

To assess the agreement of the different real-time maps and their corresponding ML version with the final maps in terms of mean absolute error (MAE) and root mean square error (RMSE), we use the following metrics

5 $$\begin{aligned} \begin{aligned} \text {MAE}&= \frac{1}{N_{lat}N_{lon}}\sum _{lat}^{N_{lat}}\sum _{lon}^{N_{lon}} |y_{lat,lon} - {\hat{y}}_{lat,lon}|\\ \text {RMSE}&= \sqrt{\frac{1}{N_{lat}N_{lon}}\sum _{lat}^{N_{lat}}\sum _{lon}^{N_{lon}} (y_{lat,lon} - {\hat{y}}_{lat,lon})^2} \end{aligned} \end{aligned}$$

where $y_{lat,lon}$ describes a grid point of the final GIM and ${\hat{y}}_{lat,lon}$ of the real-time GIM, likewise. Both GIMs refer to the same epoch. We evaluate these metrics once for all values in the GIMs and once only for the locations with a final GIM VTEC of >80 TECU. This is to analyze the performance for the critical higher VTEC values in addition to the performance for the entire map.

In addition we quantify and compare the smoothness of different GIM products. Due to the ML image generation process a smoothing of the output GIMs is expected. To quantify whether ML GIMs are smoother than real-time or final GIMs, we calculate a metric describing the smoothness of a GIM. Therefore, we apply a Laplace filter to the GIMs, which captures local second-order intensity variations and calculate the variance of the Laplace values (Pech-Pacheco et al. 2000). If a region is smoother, it will show smaller variations. The metric is calculated for all GIMs during the test period using the variance after applying the discrete Laplace filter

6 $$\begin{aligned} \Delta _{xy}\text {VTEC}(x,y) = \begin{bmatrix} 0 & 1 & 0 \\ 1 & -4 & 1 \\ 0 & 1 & 0 \end{bmatrix}. \end{aligned}$$

#### External Jason-3 evaluation

For this evaluation, we calculate the daily bias and standard deviation of the residuals between Jason-3 and GIM VTECs for 7 different latitude bins, as in (Liu et al. 2021a). The bins are defined as ([76$^{\circ }$,50$^{\circ }$],[50$^{\circ }$,30$^{\circ }$],[30$^{\circ }$,10$^{\circ }$],[10$^{\circ }$,-10$^{\circ }$],[-10$^{\circ }$,-30$^{\circ }$],[-30$^{\circ }$,-50$^{\circ }$],[-50$^{\circ }$,-76$^{\circ }$]) in solar magnetic coordinates as ionospheric variations are tightly correlated with solar and magnetic activity. The formulas to estimate bias and standard estimation are

7 $$\begin{aligned} \begin{aligned} \mu&= \frac{1}{N}\sum _{i=1}^N\text {VTEC}_\text {GIM,i}-\text {VTEC}_\text {Jason-3,i} \\ \text {STD}&= \sqrt{\frac{1}{N-1}\sum _{i=1}^N((\text {VTEC}_\text {Jason-3,i}+\mu )-\text {VTEC}_\text {GIM,i})^2} \,. \end{aligned} \end{aligned}$$

The standard deviation is a measure of how well the GIMs and the external satellite altimetry data agree over oceanic regions. The bias can be compared among different GIM products to analyse their consistency. The VTEC data from the GIM needs to be interpolated to the location of the satellite altimetry observation, which we do by cubic grid interpolation (Virtanen et al. 2020).

#### External SPP evaluation

For every GNSS station, we calculate different SPP solutions using only GNSS observations at the L1 band with corrections from different GIM products using the in-house PPPx software. This single-frequency kinematic positioning solution is then compared to a static dual-frequency solution, which serves as a reference for comparison. Coordinates are transformed into a local system with East (E), North (N) and Up (U) components. The impact of the different GIM products is then evaluated for each component as well as for the 3D position. For the individual components, we calculate the MAE between the dual-frequency and the single-frequency solution. The 3D error is calculated as Euclidean distance $dist = \sqrt{\Delta N^2+\Delta E^2+\Delta U^2}$. The configuration of the SPP processing is highlighted in Table 2.

**Table 2:** Processing details for SPP solutions

Category	Setting
Positioning mode	Kinematic
GNSS constellation	GPS + Galileo
Sampling rate	30 s
Elevation mask	7$^{\circ }$
Satellite orbit	Broadcast product from IGS
Satellite clock	Broadcast product from IGS

## Results

In this section, we present the numerical and visual results obtained using the evaluation methods described in Sect. 3.5 for both the CNN and cGAN methods.

### Internal GIM evaluation

Table 3 contains the MAE and RMSE results for the datasets with the IGS final GIMs as reference and target, whereas Table 4 contains the results for the dataset with the UPC final GIMs as reference. The results show that for all three datasets ML is able to improve the consistency of the classical real-time product towards the final GIM product. The largest improvement can be found for the IGS real-time GIMs, which also show the largest discrepancy compared to the IGS final GIMs. Here, the ML GIMs improve the MAE by 36%. Especially for the higher VTEC values above 80 TECU (see Fig. 4 for the distribution of VTEC values), the ML real-time GIMs reduce the MAE by more than 5 TECU, which is an improvement of nearly 50%. We further observe that the UPC real-time GIMs shows smaller differences compared to the IGS final GIMs than the IGS real-time GIMs. The least improvement can be found for the UPC-UPC dataset (see Table 4) with around 0.35 TECU, which still is an improvement of 14%.

The MAE of cGAN and CNN are very similar, with a difference of approximately 0.1 TECU when considering all grid points. For the VTEC values above 80 TECU, cGAN achieves a slightly lower MAE than the CNN for all three datasets.

Since an ensemble of models is used, we also calculate the STD of the predictions among the different models (see Table 5) that reflects the epistemic uncertainty. Among the different datasets and methods, we find standard deviations ranging from 1.10 to 1.66 TECU. As expected, the standard deviation is smaller for datasets where the difference between the real-time product and the final product is initially smaller. For all datasets, we observe that the standard deviation of the cGAN is higher than that of the CNN, which is likely due to the additional randomness of the discriminator, also influencing the generator training. The generally high standard deviation among the models might be due to the different distribution of training and test data. As mentioned in section 2, the test set covers a high solar activity period while only a small part of the training data is of similarly high solar activity. This difference in distributions, also shown in Fig. 4, poses an additional difficulty to the model.

**Table 3:** Results of the internal GIM evaluation for the IGS-IGS and UPC-IGS datasets

Product	MAE [TECU]	RMSE [TECU]	MAE for VTEC > 80	RMSE for VTEC > 80
IGS real-time	3.60	4.86	10.50	12.80
**IGS-IGS CNN**	**2.30**	**3.16**	**5.47**	**7.06**
**IGS-IGS cGAN**	**2.30**	**3.17**	**5.30**	**6.90**
UPC real-time	2.85	3.81	6.74	8.51
**UPC-IGS CNN**	**2.03**	**2.75**	**4.45**	**5.79**
**UPC-IGS cGAN**	**2.19**	**2.94**	**4.37**	**5.62**

ML GIMS results are bolded for better visual distinction

Benchmark for the comparison are the IGS final GIMs. Bold rows mark the ML GIMs.

**Table 4:** Results of the internal GIM evaluation for the UPC-UPC dataset

Product	MAE [TECU]	RMSE [TECU]	MAE for VTEC > 80	RMSE for VTEC > 80
UPC real-time	2.41	3.32	5.34	7.18
**UPC-UPC CNN**	**2.06**	**2.83**	**4.64**	**6.15**
**UPC-UPC cGAN**	**2.07**	**2.86**	**4.11**	**5.54**

ML GIMS results are bolded for better visual distinction

Benchmark for the comparison are the UPC final GIMs. Bold rows mark the ML GIMs.

**Table 5:** Standard deviation among the models in the ensemble for the different datasets and methods

Product	Ensemble STD [TECU]	
	CNN	cGAN
IGS-IGS	1.42	1.66
UPC-IGS	1.12	1.56
UPC-UPC	1.10	1.42

**Fig. 9:**
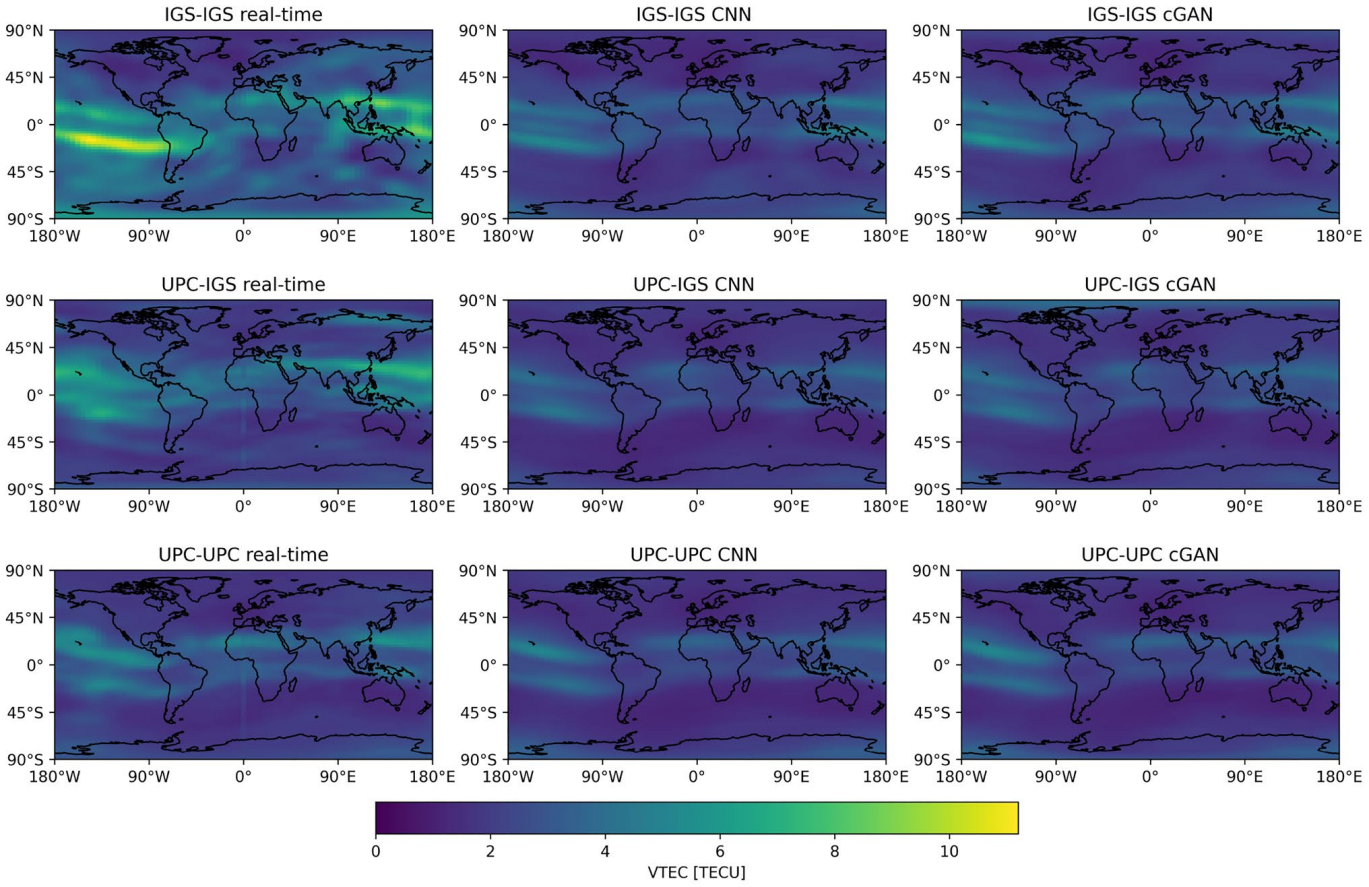
Mean absolute differences over the test period of classical real-time GIMs (left) and both CNN (middle) and cGAN GIMs (right) compared to the corresponding final GIM. Each row corresponds to one of the three datasets (IGS-IGS, UPC-IGS and UPC-UPC)

We further visualize the effect of the applied machine learning models with the mean absolute differences of the real-time products compared to the final products during the test period. Figure 9 shows these differences for the three datasets. A clear reduction of the error patterns can be seen on a global scale. Furthermore, the error in the equatorial ionozation anomaly (EIA) is also visibly reduced in all examples. The hemisphere asymmetry visible in the IGS real-time GIMs with especially lower accuracy in the southern hemisphere is also clearly reduced by the ML methods.

#### Geomagnetic storm

The ionosphere can be affected by disturbances in the geomagnetic field, known as geomagnetic storms, which lead to drastic variations of the total electron content (Mondal et al. 2024). This section provides information about the agreement of different real-time GIMs with the final GIMs for the most extreme storm day of our test period. The storm date is 2023-08-05 with a Disturbance Storm Time (Dst) index of -93 nT. Results are presented in Tables 6 and 7 and indicate that both the CNN and cGAN models are able to reduce the MAE of the classical real-time GIMs. Additionally, it seems that the cGAN GIMs achieve a slightly better performance for high VTEC values on this geomagnetic storm day compared to the CNN GIMs.

**Table 6:** Mean absolute errors between classical real-time GIMs and ML real-time GIMs compared to the final GIM during the 2023-08-05 geomagnetic storm

Product	MAE [TECU]	RMSE[TECU]	MAE for VTEC > 80	RMSE for VTEC > 80
IGS real-time	3.68	5.14	14.38	16.19
**IGS-IGS CNN**	**2.61**	**3.46**	**5.71**	**7.31**
**IGS-IGS cGAN**	**2.41**	**3.21**	**4.90**	**6.56**
UPC real-time	3.08	4.35	10.41	12.52
** UPC-IGS CNN**	**2.33**	**3.28**	**6.34**	**7.67**
** UPC-IGS cGAN**	**2.39**	**3.32**	**5.92**	**7.37**

ML GIMS results are bolded for better visual distinction

Results are for the IGS-IGS and UPC-IGS datasets.

**Table 7:** Mean absolute errors between classical real-time GIMs and ML real-time GIMs compared to the final GIM during the 2023-08-05 geomagnetic storm

Product	MAE [TECU]	RMSE[TECU]	MAE for VTEC >80	RMSE for VTEC >80
UPC real-time	2.82	3.89	8.22	10.47
**UPC-UPC CNN**	**2.36**	**3.29**	**8.14**	**9.84**
**UPC-UPC cGAN**	**2.31**	**3.20**	**6.09**	**7.61**

ML GIMS results are bolded for better visual distinction

Results are for the UPC-UPC dataset.

#### Smoothness of different GIMs

**Fig. 10:**
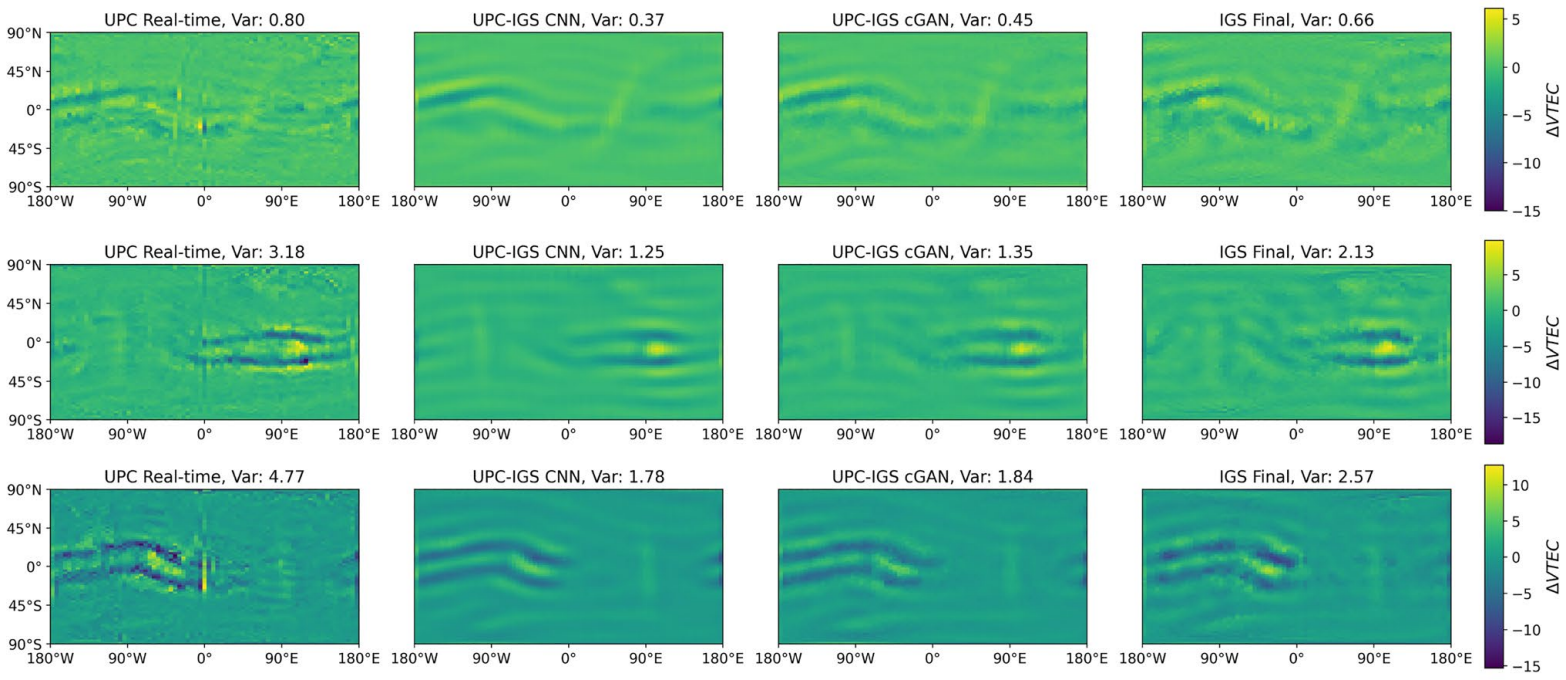
Laplace filter applied to three sets of real-time, CNN, cGAN and final GIMs of the UPC-IGS dataset during the test period. (Top) 2023-07-03 02:00:00 UT, (middle) 2023-09-23 12:15:00 UT, (bottom) 2023-10-03 23:15:00 UT. Var is the variance of the Laplace values of each GIM

We calculate the introduced metric, describing the smoothness as variance of a Laplace filtered GIM, for all GIMs during the test period and present the values in Table 8. It shows that all ML GIMs show lower variances of Laplace-filtered values than the real-time and final GIMs and therefore are smoother. This is a common and expected effect when applying ML to images and working with L1 or L2 losses (Pathak et al. 2016). The variances for the cGAN GIMs are slightly higher than for the CNN GIMs which shows that they are less affected by this effect. A visualization of a Laplace filter applied to a set of GIMs is presented in Fig. 10. It shows the real-time, CNN, cGAN and final GIM of the UPC-IGS dataset for three different epochs during the test period. The real-time GIMs show the largest variance of Laplace values, which might also indicate the presence of noise in the GIM. The CNN and cGAN GIMs show the lowest Laplace variance and appear visually as the smoothest. The variance of both types of ML GIMs does not fully reach the variance of the final GIMs.

**Table 8:** Global variance of the Laplace-filtered GIMs ($\Delta \text {VTEC[TECU]}$) for the test period

Dataset	real-time GIM	CNN GIM	cGAN GIM	Final GIM
IGS-IGS	1.54	**0.89**	**0.98**	1.53
UPC-IGS	2.23	**0.97**	**1.09**	1.52
UPC-UPC	2.23	**0.98**	**1.46**	2.98

ML GIMS results are bolded for better visual distinction

### External Jason-3 evaluation

The comparison results against satellite altimetry are listed in Table 9, which shows the average daily biases and standard deviations of the residuals for each latitude bin. It shows that the final GIMs generally have a higher bias to Jason-3 VTEC than the real-time GIMs. Additionally, the biases of the CNN and cGAN GIMs are closer to those of the final GIMs than the original real-time GIMs. This is an indication that the generated ML GIMs are more consistent with the final GIMs. For the lower-latitude bins, which correspond to regions with higher VTEC values, the estimated biases are larger than those observed at higher latitudes. Regarding the estimated standard deviations, the final GIMs typically show the lowest values. Similarly, the highest STD values occur in low-latitude regions with higher VTEC values and stronger variations. The IGS-IGS ML GIMs show lower standard deviations than the IGS real-time GIMs. However, for the UPC-IGS and UPC-UPC datasets, the standard deviations of the ML GIMs are slightly higher than those of the UPC real-time GIMs. Additionally, in certain latitude bins such as [30$^{\circ }$,10$^{\circ }$] and [–10$^{\circ }$,–30$^{\circ }$], the UPC real-time GIMs exhibit even lower standard deviations than the UPC final GIMs. The STD for the UPC-IGS and UPC-UPC ML GIMs is only slightly higher, but it contradicts the internal evaluation, which showed that ML GIMs align more closely with the final GIMs.

To further analyze this discrepancy, we calculate the differences and their STD between the final GIMs and ML GIMs, as well as between the final GIMs and real-time GIMs along the Jason-3 tracks. Tables 10, 11 and 12 show these results for the three datasets, respectively. For all the datasets, the ML GIMs have a lower STD relative to the final GIMs than the classical real-time GIMs. This shows that the ML GIMs VTEC values align better with the final GIMs VTECs along the Jason-3 groundtracks. Especially in the low latitude bins the effect is evident. Although this confirms that the machine learning approach works as intended, it does not necessarily imply better agreement with Jason-3 VTEC. We hypothesize that the higher standard deviation of the ML GIMs in this Jason-3 evaluation results from their smoother structures, which may not capture the high-frequency variations recorded by Jason-3. This is an effect of the characteristics described in Sect. 4.1.2.

**Table 9:** Biases and standard deviations of different GIM product versus Jason-3 VTEC for the test period

Product	[76$^{\circ }$,50$^{\circ }$] (Bias/STD)	[50$^{\circ }$,30$^{\circ }$] (Bias/STD)	[30$^{\circ }$,10$^{\circ }$] (Bias/STD)	[10$^{\circ }$,-10$^{\circ }$] (Bias/STD)	[-10$^{\circ }$,-30$^{\circ }$] (Bias/STD)	[-30$^{\circ }$,-50$^{\circ }$] (Bias/STD)	[-50$^{\circ }$,-76$^{\circ }$] (Bias/STD)
IGS Final	4.91/1.98	6.61/3.18	7.63/5.64	8.57/4.98	6.18/5.07	5.22/2.53	3.66/2.92
UPC Final	4.37/2.07	5.47/3.42	4.38/6.94	5.49/5.17	5.18/5.88	4.76/2.54	3.71/3.08
IGS real-time	3.91/2.58	5.45/4.50	4.14/7.73	4.68/6.64	2.16/7.54	2.72/4.20	0.85/4.55
UPC real-time	3.10/2.55	4.19/3.62	3.00/6.77	5.23/5.45	4.40/5.64	4.36/2.69	3.45/3.35
**IGS-IGS CNN**	**4.99/2.45**	**5.83/3.82**	**5.90/6.71**	**7.47/5.97**	**4.36/5.83**	**4.19/3.06**	**2.49/3.57**
** IGS-IGS cGAN**	**5.03/2.48**	**5.93/3.92**	**6.17/6.79**	**7.65/6.05**	**4.50/5.89**	**4.34/3.03**	**2.69/3.55**
**UPC-IGS CNN**	**4.44/2.65**	**6.26/3.78**	**7.16/6.68**	**8.18/5.67**	**5.58/5.93**	**4.83/2.86**	**3.23/3.41**
** UPC-IGS cGAN**	**3.98/2.69**	**6.41/3.89**	**7.25/6.76**	**8.26/5.75**	**5.71/6.00**	**5.00/2.89**	**3.39/3.49**
** UPC-UPC CNN**	**3.86/2.66**	**4.84/3.74**	**3.91/7.09**	**5.18/5.63**	**4.38/6.34**	**4.27/2.76**	**3.25/3.49**
** UPC-UPC cGAN**	**3.96/2.78**	**5.18/3.87**	**4.50/6.95**	**5.72/5.73**	**4.63/6.40**	**4.37/2.72**	**3.43/3.40**

ML GIMS results are bolded for better visual distinction

Evaluation is separated into different solar magnetic latitude bins. Latitude values are given in solar magnetic coordinates.

**Table 10:** Standard deviation of VTEC from different GIM products of the IGS-IGS dataset versus the IGS final GIM in TECUs evaluated along the Jason-3 groundtracks for the test period

Product	[76$^{\circ }$,50$^{\circ }$] (STD)	[50$^{\circ }$,30$^{\circ }$] (STD)	[30$^{\circ }$,10$^{\circ }$] (STD)	[10$^{\circ }$,-10$^{\circ }$] (STD)	[-10$^{\circ }$,-30$^{\circ }$] (STD)	[-30$^{\circ }$,-50$^{\circ }$] (STD)	[-50$^{\circ }$,-76$^{\circ }$] (STD)
IGS real-time	1.86	3.14	5.41	5.02	5.53	3.38	3.35
**IGS-IGS CNN**	**1.74**	**2.60**	**4.05**	**3.63**	**3.40**	**1.91**	**2.11**
**IGS-IGS cGAN**	**1.80**	**2.68**	**4.14**	**3.63**	**3.48**	**1.87**	**2.07**

ML GIMS results are bolded for better visual distinction

**Table 11:** Standard deviation of VTEC from different GIM products of the UPC-IGS dataset versus the IGS final GIM in TECUs evaluated along the Jason-3 groundtracks for the test period

Product	[76$^{\circ }$,50$^{\circ }$] (STD)	[50$^{\circ }$,30$^{\circ }$] (STD)	[30$^{\circ }$,10$^{\circ }$] (STD)	[10$^{\circ }$,-10$^{\circ }$] (STD)	[-10$^{\circ }$,-30$^{\circ }$] (STD)	[-30$^{\circ }$,-50$^{\circ }$] (STD)	[-50$^{\circ }$,-76$^{\circ }$] (STD)
UPC real-time	1.84	2.68	4.65	4.03	3.60	2.23	2.33
**UPC-IGS CNN**	**1.79**	**2.45**	**3.81**	**3.34**	**2.92**	**1.62**	**1.87**
**UPC-IGS cGAN**	**1.90**	**2.63**	**3.89**	**3.38**	**2.94**	**1.72**	**2.06**

ML GIMS results are bolded for better visual distinction

**Table 12:** Standard deviation of VTEC from different GIM products of the UPC-UPC dataset versus the UPC final GIM in TECUs evaluated along the Jason-3 groundtracks for the test period

Product	[76$^{\circ }$,50$^{\circ }$] (STD)	[50$^{\circ }$,30$^{\circ }$] (STD)	[30$^{\circ }$,10$^{\circ }$] (STD)	[10$^{\circ }$,-10$^{\circ }$] (STD)	[-10$^{\circ }$,-30$^{\circ }$] (STD)	[-30$^{\circ }$,-50$^{\circ }$] (STD)	[-50$^{\circ }$,-76$^{\circ }$] (STD)
UPC real-time	1.63	2.66	5.20	3.95	3.67	1.94	2.24
**UPC-UPC CNN**	**1.58**	**2.26**	**4.10**	**3.20**	**2.94**	**1.54**	**1.95**
**UPC-UPC cGAN**	**1.63**	**2.28**	**3.99**	**3.26**	**2.98**	**1.56**	**1.99**

ML GIMS results are bolded for better visual distinction

### External SPP evaluation

**Fig. 11:**
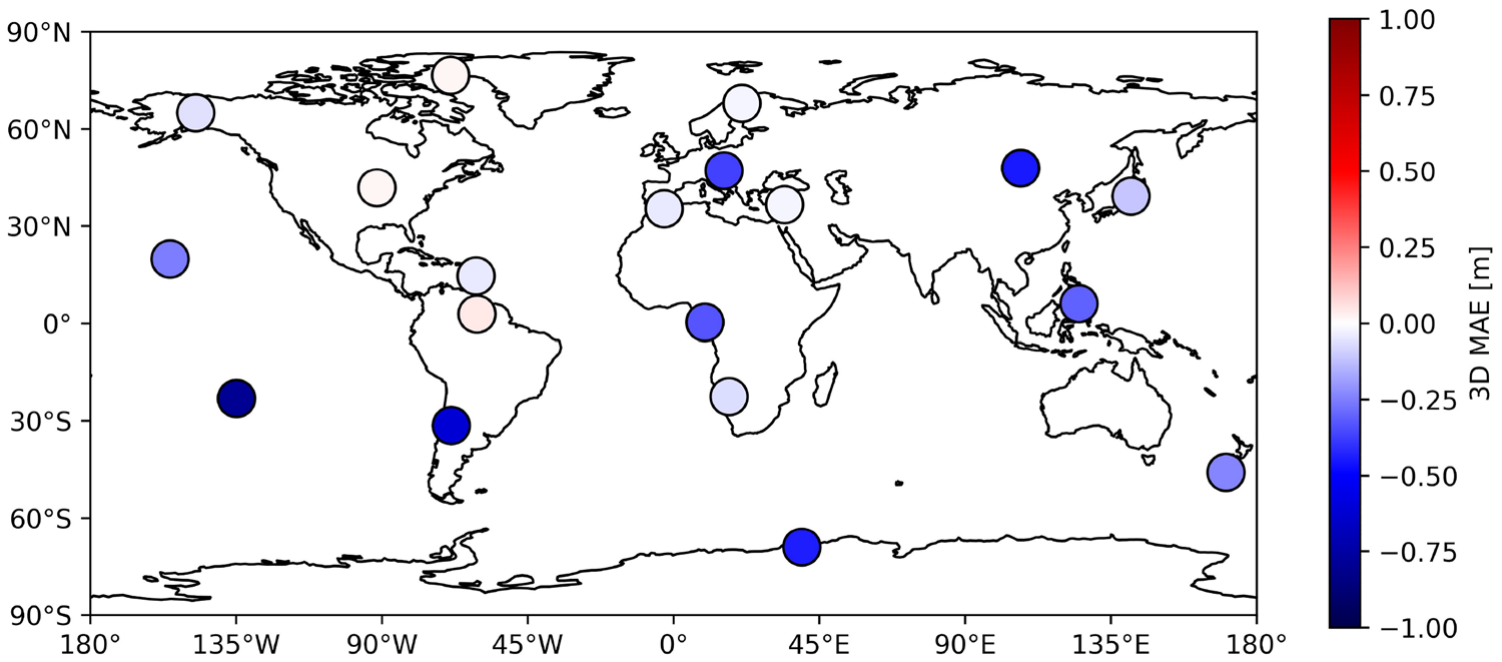
Map representing the change in 3D MAE for SPP by using the IGS-IGS CNN GIMs instead of the IGS real-time GIMs for the 19 test stations. Blue colors represent an improved positioning accuracy (less 3D MAE) by using the ML GIMs

Table 13 shows the results of the SPP evaluation for all GIM products during the test period. The solutions created by using the ML GIM corrections exhibit a lower error than the classical real-time GIMs for the IGS-IGS and UPC-IGS dataset, indicating the positive impact of the ML GIMs on positioning. For the IGS-IGS dataset we find mean improvements around 0.21 m for the 3D MAE by using the ML GIMs. For the UPC-IGS dataset the mean improvement is only around 0.07 m and for the UPC-UPC dataset we observe the same 3D MAE for ML and classical real-time product. For the UPC-UPC the results also show that the Up component is improved by ML but the accuracy of the East and North components decreases. Similar to the evaluation with satellite altimetry, the lowest errors are observed when using final GIMs, which is expected. Between the CNN and cGAN method, no clear preference is visible. Overall, this evaluation demonstrates that refining the classical real-time GIMs with machine learning leads to a real-time product that enables improved positioning by achieving smaller errors when compared to a dual-frequency solution. The largest effect is observed in the Up component, while the East component shows the least. Among all the real-time products in Table 13 the IGS-IGS ML GIMs exhibit the lowest positioning errors. A visualization of the impact on positioning performance by using the IGS-IGS CNN GIM product instead of the real-time IGS GIM is shown in Fig. 11.

**Table 13:** Positioning errors of single point positioning solutions obtained by the use of different global ionospheric map products compared to a dual-frequency solution

Product	East MAE [m]	North MAE [m]	Up MAE [m]	3D MAE [m]
IGS Final	0.437	0.550	1.240	1.579
UPC Final	0.479	0.668	1.310	1.721
IGS real-time	0.495	0.749	1.561	1.979
UPC real-time	0.482	0.681	1.473	1.869
**IGS-IGS CNN**	**0.477**	**0.668**	**1.335**	**1.760**
**IGS-IGS cGAN**	**0.480**	**0.670**	**1.350**	**1.758**
**UPC-IGS CNN**	**0.494**	**0.673**	**1.357**	**1.775**
**UPC-IGS cGAN**	**0.503**	**0.679**	**1.380**	**1.803**
**UPC-UPC CNN**	**0.512**	**0.734**	**1.419**	**1.866**
**UPC-UPC cGAN**	**0.511**	**0.724**	**1.411**	**1.854**

ML GIMS results are bolded for better visual distinction

## Conclusions and outlook

In this study, we investigated the suitability of two different ML algorithms, namely CNN and cGAN, to improve the accuracy of real-time GIMs. We used real-time and final GIMs from IGS and UPC and trained the models on a 2.5 (IGS) and 3.5 (UPC) year period to learn the deficiencies of the real-time GIMs compared to the final GIMs. The proposed methods and the corresponding models were evaluated over a 3.5 month test period during high solar activity. We compared the classical real-time GIMs and their corresponding ML-enhanced version with the final GIMs to measure the improvement in accuracy due to the application of ML. We find that ML shows excellent capabilities in correcting the real-time GIMs towards the more accurate final GIMs. For the IGS real-time GIM, we witness an enhancement of 36% in overall accuracy, with an even more substantial improvement of 47% for VTEC values exceeding 80 TECU. For the UPC real-time GIM, we still see an improvement of 26% towards the IGS final GIM and 14% towards the UPC final GIM. The ML models appear to have a stronger impact on real-time GIMs that show a larger error compared to the final GIMs, meaning it is especially beneficial for real-time GIMs with lower accuracy. To evaluate the performance of the different GIM products with external data, we compared the VTECs over the ocean with observations from the Jason-3 satellite mission for 7 different solar magnetic latitude bins. For all datasets, the estimated altimeter biases for the ML GIMs agree better with the biases of the final GIMs than those of real-time GIMs. Regarding the standard deviation of the residuals, only for the IGS-IGS dataset the ML GIMs show lower STD than the classical real-time GIMs. For the UPC-IGS and UPC-UPC, we observe a slightly higher STD for the ML GIMs than for the real-time GIMs. We attribute this to the smoothing effect happening due to the ML processing. Evaluating the variance of Laplace-filtered GIMs, we find that the CNN and cGAN GIMs show the lowest variances, which reflects the smoothing effect. The models seem to successfully correct the real-time GIMs but to a certain extent lose some of the high-frequency details, which might contain both noise and actual signals. Since an L2 loss would further encourage this effect, we think the choice of using the L1 loss is reasonable.

To evaluate the impact of the ML-refined real-time GIMs on GNSS positioning, we performed single point positioning with the use of different GIM products (real-time, ML-based real-time and final). On average, the IGS-IGS ML GIMs improve the 3D positioning precision by approximately 0.21 m compared to the IGS real-time GIM for 19 globally distributed GNSS stations. A very similar positioning performance is observed for the UPC-IGS ML GIM, which also achieves a smaller error than the classical UPC real-time GIM. For the UPC-UPC dataset we see no change in 3D MAE between the UPC real-time GIMs and the ML versions.

We observe that both ML methods generally achieve similar results, exhibiting no clear preference for one of them. The most remarkable difference between them is the better performance of the cGAN method for higher VTEC values, which is most prominent for the geomagnetic storm. The drawback of the cGAN lies in the training process, which is more complex due to the simultaneous training of both the generator and discriminator. This involves balancing both components during training, which is not necessary for the CNN. Additionally, since the CNN only consists of a generator, the training is faster as fewer parameters need to be optimized. Overall, the IGS-IGS ML GIM seems to have the most beneficial impact in the majority of the evaluations.

All results were derived from test data collected during a period of high solar activity. Despite the training data’s bias towards lower solar activities and its incomplete coverage of a full solar cycle, the machine learning model demonstrates remarkable efficacy in enhancing classical real-time GIMs, particularly for larger VTEC values. The results during the geomagnetic storm on 2023-08-05 are also promising. However, theoretically, such extreme cases could pose challenges if their characteristic deviates substantially from the data distribution on which the model has been trained.

Our method successfully demonstrates the feasibility of refining real-time GIMs with machine learning and reducing the most prominent errors. However, capturing the small-scale variation patterns needs more dedicated investigations of other methods, which are beyond the scope of this study. The enhanced real-time GIMs could significantly aid in the task of monitoring the ionosphere in real-time, offering a more precise depiction of its current state. With a processing time on the order of milliseconds for a single GIM on a NVIDIA RTX 4070, the inference latency is negligible and makes our method perfectly suitable for real-time usage.

For future investigations, it would be interesting to directly apply ML to GNSS-based slant or vertical TEC values that are typically used to generate real-time GIMs. This approach could then be compared to existing real-time GIMs to assess the potential of a fully ML-based method without the intermediate step of utilizing an existing real-time GIM. Directly applying ML to the observed VTEC values would also theoretically not be limited by spatial resolution. This would, however, require new ML architectures as in Mao et al. (2024) since the input would no longer be image-like. Further studies using GIM data should focus on methods to better capture realistic small-scale variations.

## Data Availability

The generation of ML-enhanced real-time GIMs is performed operationally at the Geodetic Prediction Center of ETH Zurich and the related products are publicly available in the IONEX format at https://gpc.ethz.ch/Ionosphere/. Final GIM products: https://cddis.nasa.gov/archive/gnss/products/ionex/. IGS real-time GIM products: http://chapman.upc.es/irtg/. UPC real-time GIM products: http://chapman.upc.es/tomion/real-time/quick/. Solar flux data: https://services.swpc.noaa.gov/json/solar-cycle/f10-7cm-flux.json. Jason-3 data with VTEC observations: ftp://ftp.star.nesdis.noaa.gov/pub/sod/lsa/rads/data/j3. The PPPx software for SPP evaluation: https://github.com/YuanxinPan/PPPx_bin

## Appendix

See Table 14

**Table 14:** Detailed information about the IGS GNSS stations used for the SPP evaluation

Number	Station Name	Latitude	Longitude
1	LMMF	14.60	–61.00
2	BOAV	2.85	–60.70
3	MELI	35.28	–2.95
4	ULAB	47.87	107.05
5	NKLG	0.35	9.67
6	WIND	–22.58	17.09
7	GRAZ	47.07	15.49
8	MERS	36.57	34.26
9	THU2	76.54	–68.83
10	OUS2	–45.87	170.51
11	SYOG	–69.01	39.58
12	MIZU	39.14	141.13
13	PGEN	6.07	125.13
14	NLIB	41.77	–91.58
15	GCGO	64.98	–147.50
16	KIRU	67.88	21.06
17	OAFA	–31.51	–68.62
18	GAMB	–23.13	–134.97
19	MKEA	19.80	–155.46
